# Myocardial Perfusion by Coronary Computed Tomography in the Evaluation of Myocardial Ischemia: Simultaneous Stress Protocol with SPECT

**DOI:** 10.5935/abc.20190201

**Published:** 2019-12

**Authors:** Wilter dos Santos Ker, Daniel Gama das Neves, Tiago Augusto Magalhães, Alair Augusto Sarmet M. D. dos Santos, Claudio Tinoco Mesquita, Marcelo Souto Nacif

**Affiliations:** 1 Hospital Universitário Antonio Pedro, Niterói, RJ - Brazil; 2 Universidade Federal Fluminense, Niterói, RJ - Brazil; 3 Complexo Hospital de Clínicas da Universidade Federal do Paraná (CHC-UFPR), Curitiba, PR - Brazil

**Keywords:** Coronary Artery Disease/physiopathology, Myocardial Ischemia, Tomography, Emission-Computed, Single-Photon/methods, Myocardial Perfusion Imaging, Cineangiography/methods

## Abstract

**Background:**

Functional assessment to rule out myocardial ischemia using coronary computed tomography angiography (CCTA) is extremely important and data on the Brazilian population are still limited.

**Objective:**

To assess the diagnostic performance of myocardial perfusion by CCTA in the detection of severe obstructive coronary artery disease (CAD) compared with single-photon emission computerized tomography (SPECT). To analyze the importance of anatomical knowledge to understand the presence of myocardial perfusion defects on SPECT imaging that is not identified on computed tomography (CT) scan.

**Method:**

A total of 35 patients were evaluated by a simultaneous pharmacologic stress protocol. Fisher’s exact test was used to compare proportions. The patients were grouped according to the presence or absence of significant CAD. The area under the ROC curve was used to identify the diagnostic performance of CCTA and SPECT in perfusion assessment. P < 0.05 values were considered statistically significant.

**Results:**

For detection of obstructive CAD, CT myocardial perfusion analysis yielded an area under the ROC curve of 0.84 [a 95% confidence interval (CI95%): 0.67-0.94, p < 0.001]. SPECT myocardial perfusion imaging, on the other hand, showed an AUC of 0.58 (95% CI 0.40 - 0.74, p < 0.001). In this study, false-positive results with SPECT are described.

**Conclusion:**

Myocardial perfusion analysis by CTA displays satisfactory results compared to SPECT in the detection of obstructive CAD. CCTA can rule out false-positive results of SPECT.

## Introduction

In order to adequately assess coronary artery disease (CAD), both anatomical and functional analysis using myocardial perfusion methods should be considered, since both have prognostic and diagnostic value. Multimodal assessment and the combination of these techniques provide safe information on the anatomical and functional diagnosis of obstructive CAD, enabling better clinical and therapeutic planning.^1,2^

In the last years, we have observed several coronary computed tomography angiography (CCTA) studies of patients with moderate stenosis. The patients were referred to perform complementary functional tests, such as pharmacologic stress cardiac magnetic resonance imaging and single photon emission computed tomography (SPECT) to verify the presence of perfusion defects. This approach allows for, with high sensitivity and specificity, the characterization of ischemia in patients with obstructive CAD.^1-3^

Myocardial perfusion by CCTA is still little explored. Stress computed tomography (CT) myocardial perfusion imaging is a technique which has shown consistent results in the diagnosis of obstructive CAD. In its turn, myocardial perfusion scintigraphy is a well-established method for detection of CAD. The possibility of integrating anatomy and function in a single exam can enhance stratification of obstructive CAD and ensure better patient management.^3-7^

The clinical benefits of CCTA are changing the perspectives of contemporary cardiology,^7^ not only for grading stenosis, but also for characterizing the atherosclerotic load and the types of plaques. Recent data in the literature, on the evaluation of significant obstructive CAD (> 50%) by CCTA, have revealed good accuracy, with high sensitivity (82-99%) and specificity (94-98%), when compared to invasive cinecoronariography.^1-6,8^

Multicentric studies, published in the last years, have demonstrated the high negative predictive value of CCTA (95-100%), emphasizing its excellent performance in excluding CAD. This fact should be increasingly exploited in clinical practice, avoiding invasive exams.^3-6,8-10^

SPECT assessment of myocardial perfusion can allow for better stratification of patients with intermediate stenosis and definition of therapeutic strategies, aiming at better prognosis.^11-18^ On the other hand, the use of hybrid technology, which combines the anatomical information from CCTA and rubidium-82 (Rb-82) myocardial positron emission tomography (PET) perfusion imaging, presents high accuracy in CAD detection;^19-31^ however, this approach is still expensive and difficult to implement clinically.

Thus, we observe that CCTA can aggregate perfusion imaging and, therefore, be increasingly used as the initial test for CAD, which remains one of the leading causes of mortality in Brazil and worldwide. Nevertheless, although several studies have demonstrated the diagnostic and prognostic value of myocardial perfusion by CCTA in patients with suspected CAD, these data are still limited in the Brazilian population. Besides, it is uncertain whether the use of CCTA analysis can replace other myocardial perfusion methods, such as SPECT, especially in places where this method may not be available. The implementation of myocardial perfusion assessment by CCTA is simple and less expensive compared to other methods.

Our purposes were: to evaluate the diagnostic performance of myocardial perfusion assessment by CCTA for significant obstructive CAD detection compared with SPECT; to analyze the importance of anatomical knowledge to understand the presence of myocardial perfusion defects by SPECT that cannot be identified by CCTA; and to describe SPECT false positives.

## Method

This is an observational study that assessed patients clinically indicated to undergo myocardial scintigraphy for CAD stratification. All patients accepted and signed the informed consent form to participate in this research on myocardial perfusion assessment by CCTA. The study and the Free and Informed Term of Consent were approved by the Research Ethics Committee of Análise de Projetos de Pesquisa (CAPPessq), do Hospital Universitário Antônio Pedro (HUAP)/Universidade Federal Fluminense (UFF) number número 392.966.

**Figure 1:**
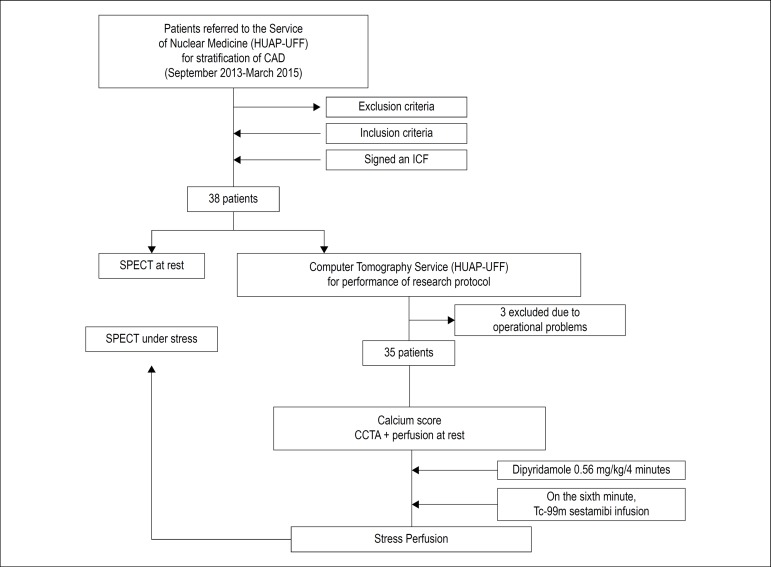
The selection of patients for this observational study included 38 patients from our institution [Antonio Pedro University Hospital – Federal Fluminense University (HUAP-UFF)], recruited in the Nuclear Medicine Service. CTA: computed angiotomography; CAD: coronary artery disease; SPECT: Single-photon emission computed tomography; ICF: Informed consent form.

Patient selection for this observational study included 38 patients from our institution [Antonio Pedro University Hospital - Federal Fluminense University (HUAP-UFF)], recruited in the Nuclear Medicine service (Figure 1).

The CCTA results (anatomy and perfusion) were considered as research data and were not reported to the patient's clinical physician, except in case of identification of significant lesions in the trunk of the left coronary artery or in the LAD coronary artery detected by CCTA. The inclusion criteria were patients with medical request for stress/rest myocardial perfusion scintigraphy to assess CAD.

Patients with creatinine above 1.5 mg/dl, obstructive pulmonary chronic disease, asthmatic patients, patients who were allergic to iodinated contrast material or for whom dipyridamole or metoprolol was contraindicated and any other aspect that the researcher deemed limiting to the method were excluded.

The exams were performed with the following flow: first the patitent was selected at the Nuclear Medicine Service and, after signing the free and informed term of consent, the patient was referred to the service of radiology to undergo CCTA (perfusion at rest) followed by myocardial perfusion under pharmacological stress with dipyridamole. Before the infusion of iodinated contrast material, during stress-induced hyperemia, 2-methoxy-isobutyl-isonitrile-99mTc (sestamibi-^99m^Tc) was infused at the computed tomography room.

The CCTA protocol included two imaging acquisitions: one for coronary anatomy assessment by CTA, which is also used to assess myocardial perfusion at rest; and a second myocardial perfusion under pharmacological stress performed shortly after the first acquisition. The mean acquisition time was 30 ± 5 minutes.

The first acquisition was volumetric and static, having been performed retrospectively using the following parameters: 120 KV, 240-400 mA and 512 × 512 matrix, 70 ml iodinated contrast media at a concentration of 350 mg/mL, infused at 5 ml/s. The second acquisition was performed following the same parameters and soon after 5 to 6 minutes from the beginning of dipyridamole infusion (Persantin^®^, Boehringer Ingelheim España S.A., España) (0.56 mg/kg/4 minutes). We chose to infuse it by hand, after images of the ascending aorta were blurred using iodinated contrast media, because it facilitates the correct selection of the beginning of acquisition, especially in the stress phase, which must occur a little earlier than usual for other coronary studies. During dipyridamole infusion, the patients’ heart rate, blood pressure and symptoms were monitored every minute. Immediately after the conclusion of stress perfusion evaluation, 240 mg of aminophylline were administered (Minoton^®^, Teuto Brasileiro S.A., Brazil) to reverse the vasodilatation effect of the stress agent. This CT protocol was idealized in a 64-detector tomographic angiography (Brilliance CT 64-slice, Philips, Netherlands) and the mean dose of radiation was 12.1 ± 5.2 mSv.

Myocardial perfusion scintigraphy (SPECT) was performed with intravenous infusion of Tc-^99m^ sestamibi, using a single-day protocol (rest-stress). The patient was referred to the Radiology Sector, and the injection of the radiotracer was performed at the tomography room, in the Radiology Sector. Soon after CT was finished, the patient was referred to stress imaging acquisition (first-passage perfusion) with a maximum interval of 30 minutes. After this stage and an interval between 60 and 120 minutes, the rest phase was performed with a new injection of Tc-99m sestamibi. The mean dose administered in each stage was 925 MBq. The images were acquired 30 to 90 minutes after intravenous administration of the agent. A total of 64 projection images of the chest were acquired from an arc of 180 degrees, from the 45-degree right anterior oblique view to the 45-degree left posterior oblique view. In the rest phase, the acquisition time was 30 seconds per projection; in the stress phase, the acquisition time was 30 seconds per projection as well. In both the stress and rest phases, ECG-synchronized image acquisition was performed.

To analyze the correlation between the myocardial perfusion techniques, the following criterion was used to characterize myocardial ischemia: there should be perfusion defects on stress images with no correspondent perfusion defect on rest images of both CCTA and SPECT.

Myocardial perfusion and CCTA were assessed visually and semi-quantitatively by two blinded and independent observers, without any knowledge of clinical data or other exams. Disagreements were resolved by means of consensus. The degree of coronary stenosis was graded, according with visual and semi-quantitative assessment by CCTA, as non-significant (< 50% reduction in luminal diameter) and significant (> 50% reduction in luminal diameter).

### Statistical Analysis

All continuous variables were expressed as mean ± standard deviation and the categorical variables as number and percentage. Fisher’s exact test was used to compare between proportions. Based on CCTA fidings, the patients were grouped according with the presence or not of significant CAD. The criterion used to define significant CAD was existence of obstruction > 50% of the lumen of coronary arteries. Sensitivity and specificity were estimated and displayed as number and percentage. The analysis of the area under the ROC curve was used to identify the efficacy of CCTA (CT perfusion) and scintigraphy (SPECT) in the diagnosis of perfusion data in this study. The research was conducted on two groups: one with stenosis > 50% on anatomical assessment by CCTA, as the "true positive" surrogate marker in this population, compared with the group with stenosis < 50% in the same method as the “true negative” (AUC ≥ 0.5 to < 0.7 = poor fit; AUC ≥ 0.7 to < 0.9 = good fit; AUC ≥ 0.9 to 1.0 = excellent fit). Intra- and interobserver agreement was obtained by using intraclass correlation coefficient reliability analysis (CCI < 0.40: poor agreement; CCI = 0.40 to 0.59: fair agreement; CCI = 0.60 to 0.74: good agreement; CCI = 0.75 to 1.00: excellent agreement). About 43% of perfusions performed using CCTA techniques (15/35) were reassessed by the same observer; the analysis was performed by a second independent observer to characterize the variability between the analyses. A total of 1,440 segments were assessed using the 16-segment model of the American College of Cardiology (ACC) and the American Heart Association (AHA), with 240 LV segments being analyzed by observer 1 at rest and, subsequently, under pharmacological stress, totaling 480 segments. Observer 1 repeated this analysis after a 3-month period, blinded to the previous analysis. Observer 2 performed the independent analysis, blind and with no previous agreement with the first observer. Both observers have more than 10 years experience in performing CCTA.

Statistical analysis was performed using MedCalc^®^ statistical software (Version 18.5 - 64-bit; MedCalc Software bvba, Ostend, Belgium). Two-tailed p values < 0.05 were considered statistically significant.

## Results

### Clinical and demographic characteristics of the sample

A total of 38 patients were selected; out of these, 35 were included in the study. Three patients were excluded: one patient due to long wait times to undergo the stress phase as a result of problems with schedule and other two due to technical problems in the Radiology Sector.

Out of the 35 patients studied, with a mean age of 52.5 ± 9 years, 18 were women (51%). Table 1 shows the main clinical and demographic characteristics of the population analysed.

**Table 1:** Clinical characteristics of the participants

Variables	Group
Age (years)	52.5 ± 9
Male sex, n (%)	17 (49)
SAH, n (%)	31 ( 88)
Diabetes Mellitus, n (%)	14 (40)
Smoking, n (%)	5 (14)
Dyslipidemia, n (%)	16 (45)
Previous AMI, n (%)	9 (26)
Typical chest pain, n (%)	10 (28)
Atypical chest pain, n (%)	8 (22)
Dyspnea, n (%)	11 (31)
Altered stress test, n (%)	1 (2)
Revascularization, n (%)	7 (20)
CAD family history, n (%)	10 (28)

SAH: Systemic arterial hypertension, AMI: Acute myocardial infarction; CAD: Coronary artery disease.

### Obstructive CAD assessment by CCTA

In this study, obstructive CAD (stenosis > 50%) was present in 43% (n = 15) of the patients; non-obstructive lesions were identified in 57% (n = 20) of the patients.

### Perfusion defects on scintigraphy and CT

**Figure 2:**
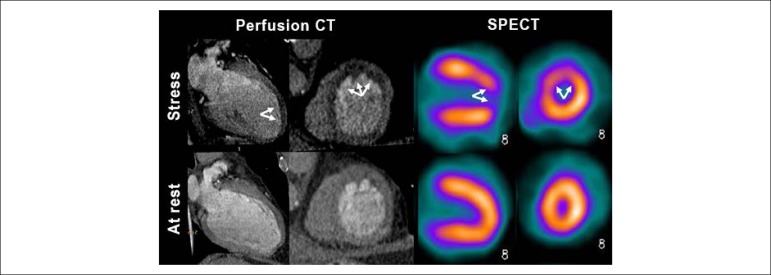
Comparison between myocardial perfusion images with stress perfusion defects on computed tomography (CT) and on single-photon emission computed tomography (SPECT). Concordant example of a same patient with significant obstructive anterior descending (LAD) coronary artery disease.

The distribution of perfusion defects on both methods are shown in Table 2. Based on the data from Table 2, it was possible to observe a difference between the distribution of perfusion defects on scintigraphy and CT. A total of 57.1% (n = 20) of the patients presented perfusion defects at myocardial scintigraphy, with only half of them (28.5%; n = 10) also presenting defects at CT. On the other hand, when perfusion defects were not detected on scintigraphy (n = 15), in the majority of the cases (60.0%; n = 9), CT showed no perfusion defects. These data showed that CT perfusion imaging sensitivity was 70%, and SPECT sensitivity was 66% for detection of perfusion defects (Figure 2).

**Table 2:** Perfusion defects on scintigraphy (SPECT) and myocardial perfusion CT (n = 35)

Perfusion defects	Positive myocardial perfusion scintigraphy	Negative myocardial perfusion scintigraphy
Positive CT myocardial perfusion	10	6
Negative CT myocardial perfusion	10	9

P = 0.73 (two-sided Fisher's exact test). SPECT: Single-photon emission computed tomography; CT: computed tomography.

### Perfusion defects on scintigraphy in relation to obstructive CAD

Based on the data in Table 3, it was possible to demonstrate a significant association between normal scintigraphy and absence of obstructive coronary lesions.

**Table 3:** Perfusion defects on scintigraphy (SPECT) and myocardial perfusion CT in relation to obstructive CAD (n = 35)

Perfusion defects	Positive SPECT *	Negative SPECT *	Positive CT **	Negative CT **
Obstructive CAD	10	5	14	1
Non-obstructive CAD	10	10	5	15

Two-sided Fisher's exact test for SPECT (*p = 0.49) and for CT (**p = 0.0001). CAD: coronary artery disease; SPECT: Single-photon emission computed tomography; CT: computed tomography.

**Figure 3:**
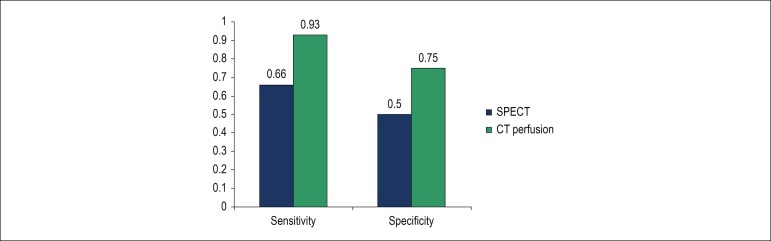
Comparison between myocardial perfusion methods sensitivity and specificity for detecting obstructive coronary artery disease. SPECT: Single-photon emission computed tomography; CT: computed tomography.

Twenty patients had abnormal myocardial scintigraphy, and half of them (n = 10) also presented obstructive CAD at CCTA. Table 4 shows false-positive scintigraphy findings. In contrast, when scintigraphy was normal (n = 15), in most of the cases (66%), there was no presence of obstructive lesions on tomography; this association did not reach statistical significance (p = 0.49). According to these data, the sensitivity of scintigraphy for anatomical assessment by CTA was 66%, with a specificity of 50% (Figure 3).

**Table 4:** False-positives on myocardial scintigraphy

Cause of false-positive	Positive SPECT	Negative SPECT
Deep myocardial bridge	2	2
Anatomical variation (short anterior descending artery)	1	1
Low levels (tracer leakage)	1	1
Patient with a 40% LAD stenosis	1	1
Patient with coronary-cavitary microfistulas	1	1
Others (microcirculation disease?)	4	4

SPECT: Single-photon emission computed tomography; CT: computed tomography.

### Perfusion defects on myocardial perfusion CT in relation to obstructive CAD

Based on the data in Table 3, it is possible to show a significant association between abnormal CT and presence of obstructive coronary lesions. Out of all the patients, 54.2% (n = 19) presented abnormal CT, and most of them (73.6%; n = 14) also presented coronary obstructive lesions on CT. In contrast, when perfusion tomography was normal, which occurred in 45.7% (n = 16) of the patients, in almost all the cases (93.7%, n = 15), the tomography showed no obstructive lesions (p = 0.0001). According to these data, CT perfusion imaging sensitivity for the diagnosis of obstructive CAD was 93%, and specificity for detecting the absence of obstructive CAD on CCTA was 75% (Figure 3).

### Analysis of the area under the curve for obstructive CAD detection

**Figure 4:**
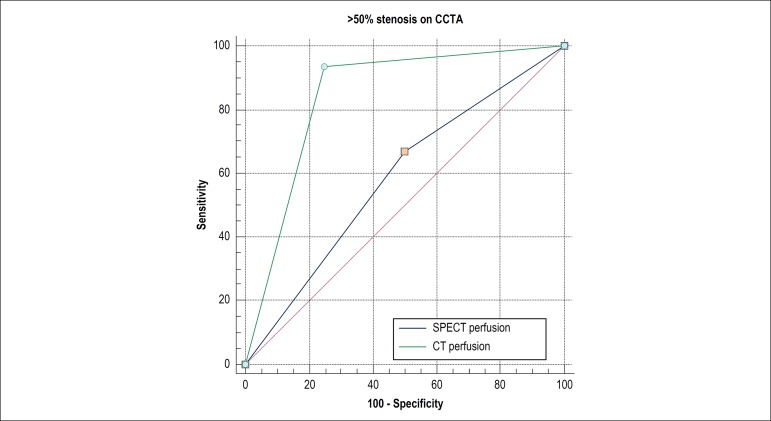
Analysis of the area under the ROC curve showing diagnostic perfusion performance of CT [0.84 (CI 95%: 0.67-0.94, p < 0.001)] and of scintigraphy (SPECT) [0.58 (CI 95%: 0.40-0.74, p < 0.001)], in this study.

Myocardial perfusion with CT showed an AUC of 0.84 for the detection of obstructive CAD, with a confidence interval (CI) range of 0.67 - 0.94 (p < 0.001). On the other hand, SPECT myocardial perfusion had an AUC of 0.58, with a CI range of 0.40 - 0.74 (p < 0.001) (Figure 4).

### Assessment of correlation between observers of computed tomography for perfusion imaging

Excellent intra- and inter observer correlation was reported in the assessment of stress perfusion, with an ICC of 0.90 (0.87-0.92) and 0.94 (0.93-0.96), respectively. The intraobserver correlation of perfusion at rest was also excellent, with an ICC of 0.96 (0.95-0.97). For interobserver correlation of perfusion at rest the result was good, with an ICC of 0.71 (0.63- 0.78).

## Discussion

In this study, it was possible to assess the diagnostic performance of myocardial perfusion by CCTA for the detection of significant obstructive CAD in relation to SPECT. The perfusion findings of scintigraphy with ^99m^Tc-sestamibi were compared with the findings of myocardial perfusion by 64-detector row computed tomography. As a strength of this study, we highlight the simultaneous use of the same pharmacological stress agent for CT perfusion image acquisition, and the administration of the radiotracer, which enables performance of CT and subsequent scintigraphy image acquisition, because it lacks significant redistribution. Another important data was the possibility for anatomical localization and correlation with the presence of myocardial perfusion defects by SPECT. In this study, it was also possible to understand why the defect was not detected by CCTA and to describe SPECT false positives.

If we assess myocardial perfusion alone, an intermediate correlation between CT and scintigraphy images will be found, especially because the sensitivity of CT perfusion sensitivity for perfusion defects detection on SPECT was 70%, with a specificity of 66%, considering that scintigraphy is the standard method used to assess perfusion. Tanami *et al*.^32^ clearly state that CCTA has better accuracy than SPECT for detecting significant obstructive CAD. Hence, it is necessary to explore this finding and understand that many patients with false-negative SPECT results are unnecessarily submitted to cardiac catheterization, due to lack of anatomical assessment.^32-35^

An interesting finding, in line with previous studies, is the comparison between the sensitivity and specificity of the two perfusion techniques in detecting obstructive coronary lesions, considering that coronary CT is the gold standard for the diagnosis of anatomic CAD.^35-37^ In this study, we observed better ischemic catheterization by CT myocardial perfusion when compared with SPECT. It is important to highlight that catheterization was not used as the gold standard and, thus, these results may vary if other methods of reference are used, such as flow fractional reserve (FFR).^37-39^ Rochitte et al.,^35^ showed that combined CCTA and stress perfusion imaging accurately identifies patients with > 50% lesion in the catheterization and who presented perfusion defects at SPECT. Moreover, the rational use of these techniques and multimodality assessment are important in modern cardiology, since they are always associated with increased exposure to radiation.^36^

In the study carried out by Arbab-Zadeh et al.,^36^ greater accuracy was observed for CT perfusion imaging when compared with SPECT (92%*versus* 62%, p < 0.001), but the authors used another methodology with a higher slice CT system (320 detectors), as well as a slightly different protocol, which is not a problem, according with recommendations.^37^ In contrast, other studies compared CCTA with SPECT and PET perfusion imaging with invasive catheterization with FFR, as a gold standard. Interestingly, perfusion PET was the exam that better correlated with the gold reference, whereas CCTA and SPECT performed similarly, showing that anatomic measures are not substitutes for functional assessment and that, even when the best method for anatomy assessment is used, functional assessment of coronary lesions is required.^36,38-40^

Another finding of the study that needs discussion is the presence of 10 patients (28%) with abnormal SPECT who did not present significant obstructive CAD on CCTA. Considering that CCTA is the anatomical method of reference in this study, we observed a high number of “false-positive” myocardial perfusion scintigraphy findings. We believe that a large part of these findings may be related with microcirculation disease (40%), since it was not possible to identify another cause that could explain them. The other findings (60%) were explained by anatomy assessment by CCTA. The best example is the case of a patient with myocardial bridge in which CT provided the anatomical substrate for the diagnosis of underlying myocardial ischemia detected by both SPECT and CT, already previously published by our research group.^41^ With regard to scintigraphy, we observed that one of the studies presented low levels of the tracer, due to tracer leakage, that was not detected during the study and, therefore, was not excluded from the analysis. We believe that further studies need to be conducted in order to better clarify these findings, because they will affect clinical decision-making.

There are several factors that can be potentially responsible for disagreements between the tests. Some of them are obvious, such as differences in spatial resolution between the techniques (CT has submillimeter resolution, whereas SPECT has a resolution of 6 mm) and the distinct contrast properties used: the ^99m^Tc-sestamibi exhibits a roll-off phenomenon, in which there is a limitation of its regional distribution when the flow is increased above certain threshold, while the same does not occur with iodinated contrast.^9,42-52^

In the Brazilian context, in spite of the absence of nuclear medicine services, combined CCTA and myocardial perfusion imaging is available, thus we consider this method as a simple and enforceable strategy. Some aspects should be considered, such as the use of beta-blockers to reduce heart rate for CCTA imaging, which can have a relative influence on the ischemic area detectable by SPECT, especially in cases of microcirculation disease. Another aspect is obesity, because in these patients the quality of the images is worsened, which can cause disagreement between the techniques. Another point is that, in order to perform CT perfusion, the patient needs to be inside the equipment in the stress phase, which makes the use of pharmacological stress mandatory. If physical stress could be used, perhaps the results would have been different from what we found.^35,42,44^

For CCTA, undoubtedly, the greatest limitation is exposure to radiation and iodinated contrast media, which are agents with potential adverse events. This protocol optimization, with new equipment, may be capable of reducing the levels of exposure; however, even so, the protocol shall only be adopted in selected patients, where information can be complemented. Studies using 320 detectors have shown that the combination of CT perfusion and CCTA can promote lower radiation exposure compared to the conventional protocol for myocardial perfusion imaging (9 mSv and 13 mSv, respectively).^35,36^

Standardization of CT analysis is still a limitation, and the use of automatic analysis software is one of the priorities for technology development, since there are no polar maps yet, as in nuclear medicine, to display ischemic and normal patients for quantification of the level of ischemia, with validated and widely available software.

Among other limitations of our study, as we detailed throughout the discussion, is the small number of individuals recruited. We believe that this is a partial limitation and should encourage further studies in different populations. We also took into account the false-positive scintigraphy results that might have influenced its performance, because we believe that the majority of cases can be explained by anatomy. Last but not least, one could imagine that the use of CCTA as an anatomical test would be limiting. In this case, numerous studies have compared CTA and catheterization with excellent results, which validates this approach.

## Conclusion

Myocardial perfusion assessment by CCTA, after dipyridamole stress, is feasible and simple, with satisfactory results, when compared with SPECT, for obstructive CAD detection. Combined assessment of anatomy and stress perfusion by CCTA shows good capacity for detecting significant obstructive CAD, while ruling out SPECT false-positive findings.
